# Association between *KRAS* and *PIK3CA* Mutations and Progesterone Resistance in Endometriotic Epithelial Cell Line

**DOI:** 10.3390/cimb46040224

**Published:** 2024-04-19

**Authors:** Kosuke Kanno, Kentaro Nakayama, Sultana Razia, Sohel Hasibul Islam, Zahan Umme Farzana, Shahataj Begum Sonia, Hitomi Yamashita, Masako Ishikawa, Tomoka Ishibashi, Kayo Imamura, Tohru Kiyono, Satoru Kyo

**Affiliations:** 1 Department of Obstetrics and Gynecology, Faculty of Medicine, Shimane University, Izumo 693-0021, Japan; kanno39@med.shimane-u.ac.jp (K.K.); hasibulsohel1167@gmail.com (S.H.I.); farzanashormi99@gmail.com (Z.U.F.); sbsonia1995@gmail.com (S.B.S.); memedasudasu1103@gmail.com (H.Y.); m-ishi@med.shimane-u.ac.jp (M.I.); 2 Department of Obstetrics and Gynecology, Nagoya City University East Medical Center, Nagoya 464-8547, Japan; tomoka@med.nagoya-cu.ac.jp; 3 Department of Legal Medicine, Faculty of Medicine, Shimane University, Izumo 693-0021, Japan; raeedahmed@yahoo.com; 4 Department of Obstetrics and Gynecology, Unnan City Hospital, Unnan 699-1221, Japan; aphrodite.41.41@icloud.com; 5 Project for Prevention of HPV-Related Cancer, National Cancer Center, Exploratory Oncology Research and Clinical Trial Center (EPOC), Kashiwa 277-8577, Japan; tkiyono@east.ncc.go.jp

**Keywords:** endometriosis, progesterone resistance, *KRAS* mutation, dienogest

## Abstract

Although endometriosis is a benign disease, it is associated with cancer-related gene mutations, such as *KRAS* or *PIK3CA*. Endometriosis is associated with elevated levels of inflammatory factors that cause severe pain. In a previous study, we demonstrated that *KRAS* or *PIK3CA* mutations are associated with the activation of cell proliferation, migration, and invasion in a patient-derived immortalized endometriotic cell line, HMOsisEC10. In this study, we investigated the effects of these mutations on progesterone resistance. Since the HMOsisEC10 had suppressed progesterone receptor (PR) expression, we transduced PR-B to HMOsisEc10 cell lines including *KRAS* mutant and *PIK3CA* mutant cell lines. We conducted a migration assay, invasion assay, and MTT assay using dienogest and medroxyprogestrone acetate. All cell lines showed progesterone sensitivity with or without mutations. Regarding inflammatory factors, real-time quantitative RT-PCR revealed that the *KRAS* mutation cell line exhibited no suppression of Cox-2 and mPGES-1 on progesterone treatment, whereas IL-6, MCP-1, VEGF, and CYP19A1 were significantly suppressed by progesterone in both mutated cell lines. Our results suggest that *KRAS* mutation and *PIK3CA* mutation in endometriotic cells may not be associated with progesterone resistance in terms of aggressiveness. However, *KRAS* mutations may be associated with progesterone resistance in the context of pain.

## 1. Introduction

Endometriosis causes dysmenorrhea, chronic pelvic pain, dyspareunia, painful defecation, and infertility [1]. Its epidemiology is still controversial; however, it has been reported that 10–15% of women of reproductive age suffer from endometriosis [2,3,4,5]. Endometriosis-related pain is severe in many cases and causes school absenteeism among adolescents and has a severe impact on work productivity [6,7]. Thus, it can be concluded that this benign disease severely affects daily lives of women. Multiple mechanisms have been reported to cause pain in endometriosis [8,9]; those pain are associated with inflammatory factors such as prostaglandin, increased sensory nerve, and imbalance of neurotransmitters or spinal hyperalgesia [9]. Pain- and angiogenesis-related factors, such as Cox-2, interleukin-6 (IL-6), vascular endothelial growth factor (VEGF) [10], monocyte chemotactic protein-1 (MCP-1) [8], and cytochrome P 19A1 (CYP19A1) [11], are elevated in the endometriotic tissue. Non-steroidal anti-inflammatory drugs (NSAIDs) can reduce prostaglandin synthesis via suppression of cyclo-oxygenase-2 (Cox-2). Endometriosis is an estrogen-dependent disease [12]. Estrogens promote endometriotic cell survival and its progression or even inflammation [13], while progesterones downregulate estrogen receptors, suppress follicle-stimulating hormone (FSH) and luteinizing hormone (LH) secretion, and suppress secretion of some interleukins and angiogenesis factors [14,15]. Therefore, NSAIDs and progestins such as dienogest (DNG) or medroxyprogesterone acetate (MPA) are the major medical treatment.

Endometriosis itself is a benign disease; however, it has been reported that endometriosis has *KRAS*, *PIK3CA*, or other cancer-associated mutations [16,17,18,19]. Among ovarian cancer, *KRAS* and *PIK3CA* mutations are usually found in type 1 ovarian cancers such as low-grade serous carcinoma, mucinous carcinoma, endometrioid carcinoma (EC), and clear cell carcinoma (CCC) [20,21]. It is suggested that endometriosis is the precursor of EC and ECC and those mutations maybe associated with carcinogenesis; however, those mutations are found even in normal endometrium [22,23]. In general, those *KRAS* or *PIK3CA* mutations activate RAS/MAPK or PI3K/AKT pathways and are associated with tumor growth, proliferation, and metastasis [23], but the triggering molecular mechanisms through which *KRAS-* or *PIK3CA*-mutated normal endometrium becomes endometriosis and carcinogenesis of EC and CCC from endometriosis is still unclear. In a previous study, we established a patient-derived endometriotic cell lines and revealed that *KRAS* or *PIK3CA* mutations are associated with the activation of cell proliferation, migration, and invasion [24]. These findings suggest that patients with endometriosis with such mutations exhibit more aggressive clinical features and may be resistant to hormonal therapy [24]. Here, we analyzed whether endometriotic cell lines with *KRAS* or *PIK3CA* mutations are resistant to progesterone.

## 2. Materials and Methods

### 2.1. Progesterone Therapy Model of Endometriotic Cell Line

We modified a human immortalized endometriotic epithelial cell line we previously established [24]. In summary, we collected ovarian endometriotic cells from a patient who underwent laparoscopic cystectomy and transduced with *hTERT*, *cyclin D1*, and mutant *CDK4* (*CDK4*^R24C^) via lentivirus-mediated gene transfer [25,26], and we named this immortalized human endometriotic cell line as HMOsisEC10. Then, we established *KRAS* (*KRAS^V12^*) and *PIK3CA* (*PIK3CA^E545K^*) mutant-overexpressing cells by lentivirus vector infection. Since Western blot analysis revealed the absence of progesterone receptor (PR) expression in these cell lines, we transduced progestin receptor B (PR-B) into these cell lines using the retroviral vector pCMSCV-EM7bsd-hPRB, as previously described [24]. We designated these newly established cell lines as Vector (HMOsisEC10-PRB), HMOsisEC10KRAS-PRB, and HMOsisEC10PIK3CA-PRB. These cell lines were maintained in phenol red free DMEM with 10% dextran-coated charcoal treated (DCC)-FBS and antibiotics (50 µ/mL penicillin and 50 µg/mL streptomycin) and incubated at 37 °C in 95% air/5% CO_2_.

### 2.2. RNA Extraction and Reverse Transcriptase Polymerase Chain Reaction (RT-PCR)

Semiconfluent cells were seeded in culture flasks, harvested, and rinsed twice with cold PBS. Total RNA was extracted using the RNeasy Mini Kit (QIAGEN, Venlo, Netherlands) according to the manufacturer’s instructions, and concentrations were determined using a NanoDrop ND-1000 spectrophotometer (Thermo Fisher Scientific, Waltham, MA, USA). RT–PCR was performed using the TOYOBO RT-PCR kit (TOYOBO, Osaka, Japan). Primers used for amplification and PCR cycles were as follows: human PR-A/B common sequence forward primer 5′-CCTGACACCTCCAGTTCTTTGCTGA-3′ and reverse primer 5′-GGGATCTGCCACATGGTAAGGCATA-3′, 40 cycles; human PR-B specific upstream sequence forward primer 5′-ACACCTTGCCTGAAGTTTCG-3′ and reverse primer 5′-CTGTCCTTTTCTGGGGGACT-3′, 40 cycles; and human housekeeping gene (GAPDH) forward primer 5′-ACGGGAAGCTTGTCATCAAT-3′ and reverse primer 5′-TGGACTCCACGACGTACTCA-3′, 40 cycles. The PCR products were visualized using an AE-6962FC Light Capture (ATTO) following gel electrophoresis.

### 2.3. Western Blot Analysis

Breast cancer cell lines T47D, HMOsisEC10, HMOsisEC10-PRB, HMOsisEC10KRAS-PRB, and HMOsisEC10PIK3CA-PRB were used.

Cells were collected using a cell scraper and centrifuged. Each pellet was lysed in a lysis buffer. Subsequently, the samples were heated at 70 °C in a water bath for 10 min and cooled on ice for 1 min. LDS buffer and sample-reducing buffer were added to the cooled samples and were centrifuged at 150 rpm for 5 min. The samples were then separated using sodium dodecyl sulfate-polyacrylamide gel electrophoresis (Invitrogen, Carlsbad, CA, USA) and transferred to polyvinylidene fluoride membranes using Bio-Rad semi-dry trans blotters (Trans-Blot^®^ SD cell) (BIO-RAD, Hercules, CA, USA). The membranes were blocked with LI-COR blocking buffer (LI-COR, Lincoln, NE, USA) for 1 h at room temperature. After 1 h, the membranes were incubated with primary antibodies (Supplementary Table S1), diluted in LI-COR blocking buffer containing 0.1% Tween, overnight on a shaker at 4 °C. Following incubation, the membranes were washed four times for 5 min each with TBST and probed with secondary antibodies (goat anti-mouse or goat anti-rabbit IR-Dye 670 or 800 CW-labeled) for 1 h. These membranes were washed four times for 5 min each in TBST, followed by addition of TBS, and imaged using a LI-COR Odyssey scanner (LI-Odyssey Infrared Imaging System, ICW, Lincoln, NE, USA). Boxes were manually regulated over each band of interest and near-infrared fluorescent values for raw intensity—with intra-lane background subtracted—were obtained using Odyssey 3.0 analytical software (Model-9120, S/N: ODY-2280, LI-COR, Lincoln, NE, USA).

### 2.4. Dienogest (DNG) and Medroxyprogesterone Acetate (MPA) Therapy

For assessing the effects of DNG and MPA on each cell line, we conducted MTT assays using several concentrations of DNG and MPA. We found 15 μM to be the most stable concentration. Therefore, 15 μM DNG (Abcam plc, Cambridge, UK) was added to the DNG group, and 15 μM MPA (Medchemexpress, Deer Park Dr, NJ, USA) was added to the MPA group.

### 2.5. Migration Assay

We conducted a wound healing assay to assess migration ability. Cells were seeded in 6-well culture plates at a density of 1 × 10^6^ cells/well and grown to 90–100% confluent monolayers. The cell surface was scraped by a 200-microliter pipette tip to create an acellular area. The plates were then gently washed twice with culture medium to remove non-attached cells. The rate of defect closure was measured by monitoring wound healing for 24 h. Individual cells in the wound area were quantified as the average of multiple fields at 200× magnification.

### 2.6. Matrigel Invasion Assay

Corning BioCoat Matrigel Invasion Chamber (Discovery Labware Inc., Bedford, MA, USA) with an 8 microns pore size was used for the invasion assay. Serum-free medium (500 μL) was added to the upper and bottom chambers and incubated in a humidified tissue culture incubator at 37 °C with 5% CO_2_ for 1–2 h. Subsequently, the serum-free medium was removed from both chambers and cells were seeded at a density of 25,000/350 μL in a serum-free medium in the upper chamber. The lower chamber was filled with 900 μL of F-medium containing 20% FBS for chemoattraction. The chambers were then incubated at 37 °C under 5% CO_2_ for 24 h. After 24 h, the medium was removed from both chambers and the cells were washed twice with sterile PBS. Next, 3.7% paraformaldehyde was added to both chambers for fixation for 2 min, and 100% methanol was added to both chambers for permeabilization for 20 min. The wells were washed twice with PBS and stained with Giemsa for 15 min. Finally, the chambers were washed twice with PBS, and the uninvaded cells were gently removed using a cotton swab. Migrating cells were quantified in 16 non-overlapping fields at 200× magnification using a light microscope (BX41; Olympus, Tokyo, Japan).

### 2.7. Cell Proliferation Assay

Each cell line was seeded in a 96-well plate at a density of 3000 cells/well and subjected to MTT assay [27]. The results are expressed as the mean ± standard deviation (SD) based on triplicate replications.

### 2.8. Real-Time Quantitative PCR

The QIAGEN buffer RLT (QIAGEN GmbH, QIAGEN, Hilden, Germany) was added to cell pellets for homogenization. Total RNA was isolated according to the Qiagen standard protocol (Qiagen, Hilden, Germany). Spectrophotometry with NanoDrop ND-1000 (NanoDrop Technologies, Wilmington, DE, USA) was used to measure the RNA quantity. RT-PCR was conducted using an Applied Biosystems SYBR Green mix kit (Thermo Fisher Scientific, Waltham, MA, USA). The primers used were the same as we described before [24]. The primers used for this sequencing are summarized in Supplementary Table S2. The thermocycling profile consisted of one cycle of 95 °C for 30 s, followed by 40 cycles at 95 °C (5 s), 60 °C (30 s), and 72 °C (30 s). The 2^−ΔΔCt^ method with GAPDH levels was used to standardize gene expression levels. These experiments were independently performed at least in triplicate.

### 2.9. Statistical Analysis

Graph values are described as means ± SD of three different samples. Statistical significance was examined between the control and DNG groups or the control and MPA groups and determined using Dunnett’s test. A *p* < 0.05 was defined as statistically significant. SPSS 23.0 (SPSS Inc., Chicago, IL, USA) was used for statistical analysis.

## 3. Results

### 3.1. Western Blot Analysis and RT-PCR

**Figure 1:**
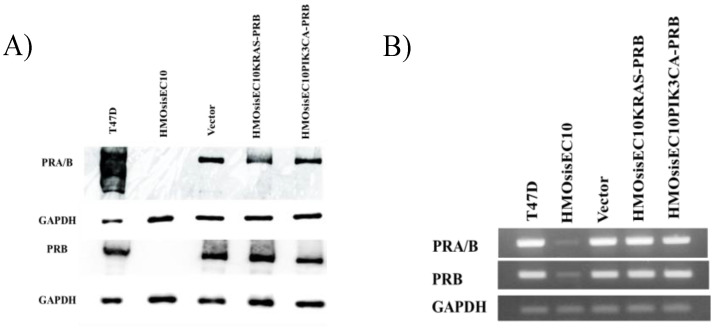
Western Blot analysis (**A**) and RT-PCR (**B**) of PR-B transduced cell lines. T47D cells exhibited PR-B expression, whereas HMOsisEC10 cells exhibited no expression in Western blotting and low expression in RT-PCR. RT-PCR revealed that Vector, HMOsisEC10 KRAS-PRB, and HMOsisEC10PIK3CA-PRB demonstrated PR-B expression.

Western blot analysis revealed that HMOsisEC10 cells did not express PR-A or PR-b, whereas HMOsisEC10-PRB, HMOsisEC10KRAS-PRB, and HMOsisEC10PIK3CA-PRB cells expressed PR-B (Figure 1).

### 3.2. Migration, Invasion, and Proliferation Assay

**Figure 2:**
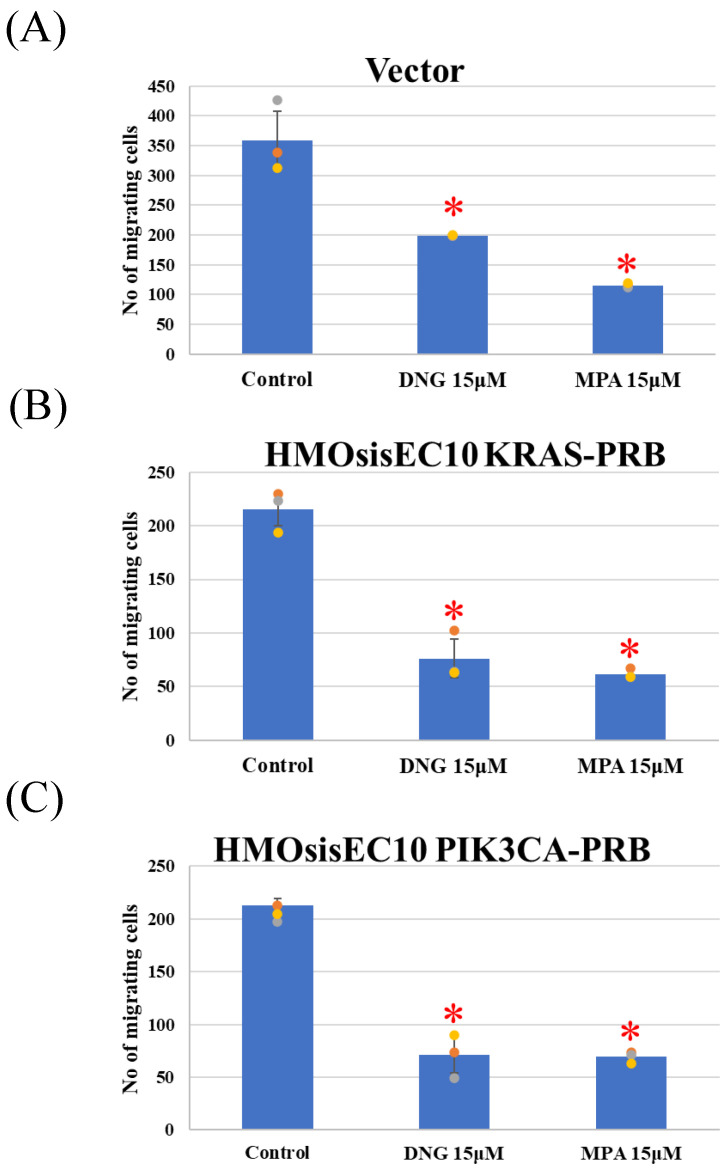
Migration assay using (**A**) Vector, (**B**) HMOsisEC10KRAS-PRB, and (**C**) HMOsisEC10PIK3CA-PRB cells. The Vector, HMOsisEC10KRAS-PRB, and HMOsisEC10PIK3CA-PRB cells demonstrated significant inhibition of migration under either dienogest (DNG) or medroxyprogesterone acetate (MPA). * *p* < 0.05 examined using Dunnett’s test. n = 3. The error bars indicate standard deviation.

**Figure 3:**
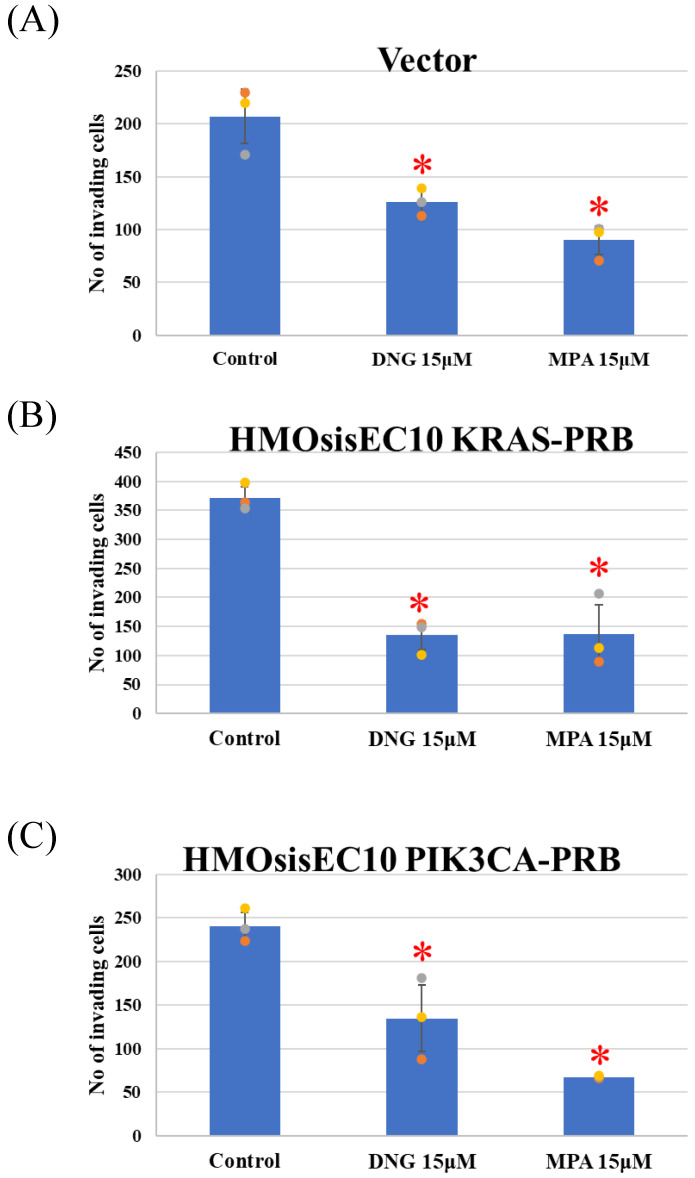
Invasion assay using (**A**) Vector, (**B**) HMOsisEC10KRAS-PRB, and (**C**) HMOsisEC10PIK3CA-PRB cells. The Vector, HMOsisEC10KRAS-PRB, and HMOsisEC10PIK3CA-PRB cells demonstrated significant inhibition of invasion under either dienogest (DNG) or medroxyprogesterone acetate (MPA). * *p* < 0.05 examined using Dunnett’s test. n = 3. The error bars indicate standard deviation.

**Figure 4:**
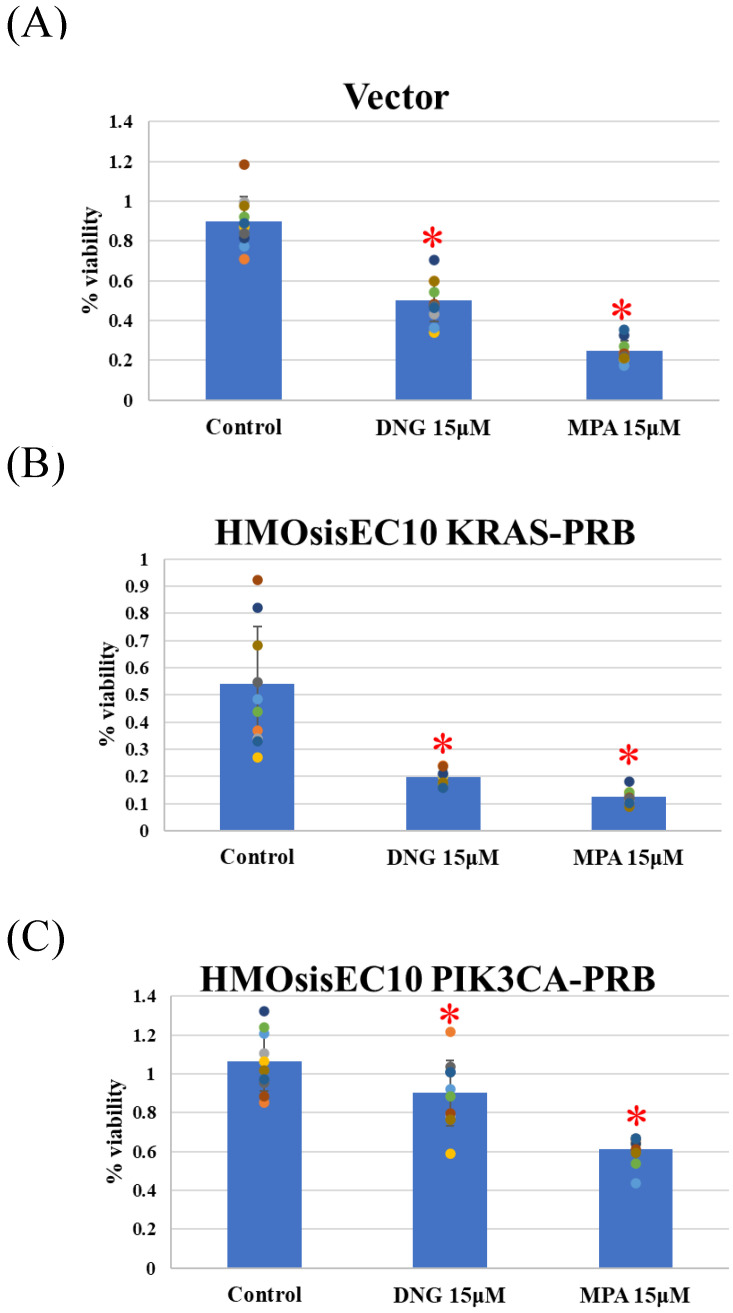
MTT assay using (**A**) Vector, (**B**) HMOsisEC10KRAS-PRB, and (**C**) HMOsisEC10PIK3CA-PRB cells. The Vector (HMOsisEC10-PRB), HMOsisEC10KRAS-PRB, and HMOsisEC10PIK3CA-PRB demonstrated significant inhibition of proliferation in the presence of either dienogest (DNG) or medroxyprogesterone acetate (MPA). * *p* < 0.05 examined using Dunnett’s test. n = 3. The error bars indicate standard deviation.

The Vector, *KRAS* mutant, and *PIK3CA* mutant cell lines showed significant inhibition of migration in the presence of DNG or MPA in the migration assay (Figure 2 and Figure S1). The matrigel invasion assay showed a significant inhibition of invasion by the Vector, and *KRAS* and *PIK3CA* mutant cell lines (Figure 3 and Figure S2). In the cell proliferation assay, the Vector, *KRAS* mutant, and *PIK3CA* mutant cell lines demonstrated inhibited proliferation in the presence of DNG or MPA (Figure 4 ).

### 3.3. Real-Time Quantitative PCR (Real-Time qPCR)

**Figure 5:**
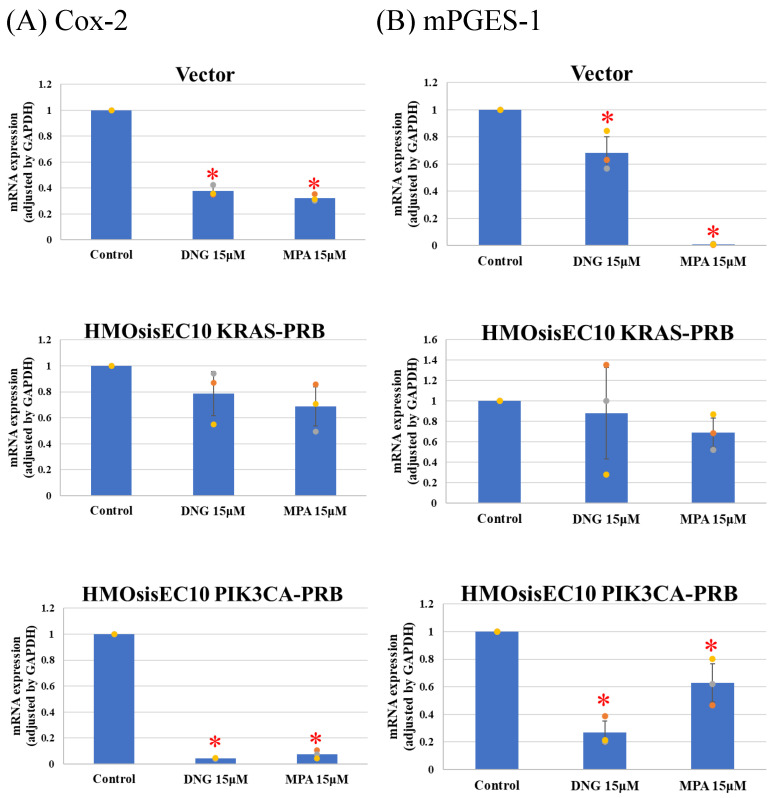
Real-Time quantitative PCR of prostaglandin synthesis-associated enzymes. Dienogest (DNG) and medroxyprogesterone (MPA) significantly suppress prostaglandin synthesis-associated enzymes in wild-type and *PIK3CA* mutant cell lines. (**A**) Cox-2 (**B**) microsomal PGE2 synthases-1 (mPGES-1). *KRAS* mutant cell line showed progesterone resistance. * *p* < 0.05 examined using Dunnett’s test. n = 3. The error bars indicate standard deviation.

**Figure 6:**
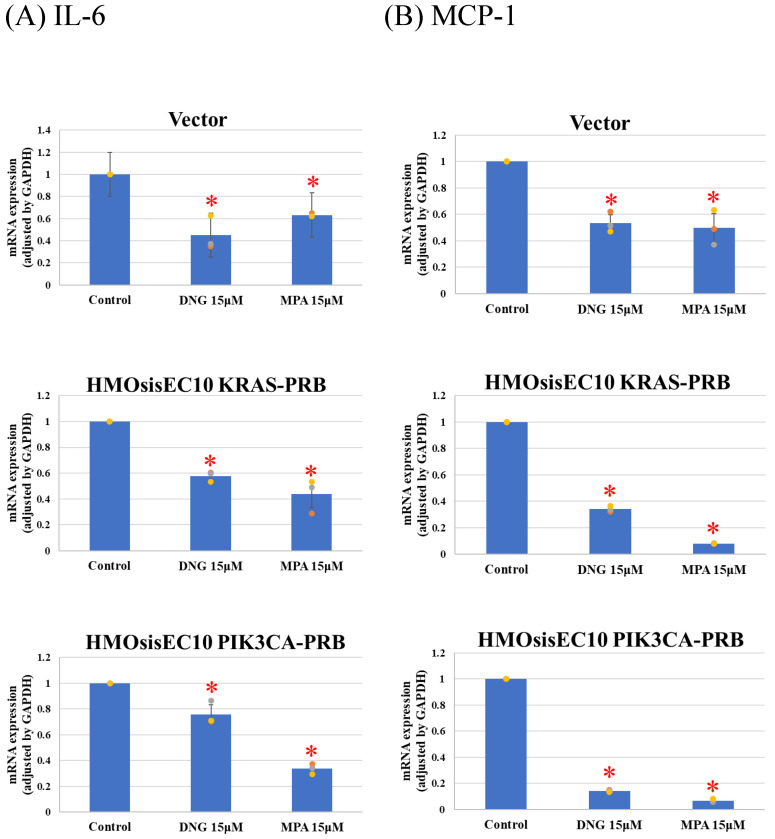
Real-Time quantitative PCR of (**A**) interleukin-6 (IL-6) and (**B**) monocyte chemotactic protein-1 (MCP-1) expression. Dienogest (DNG) and medroxyprogesterone (MPA) significantly suppress IL-6 and MCP-1 expression in mutant cell lines. * *p* < 0.05 examined using Dunnett’s test. n = 3. The error bars indicate standard deviation.

**Figure 7:**
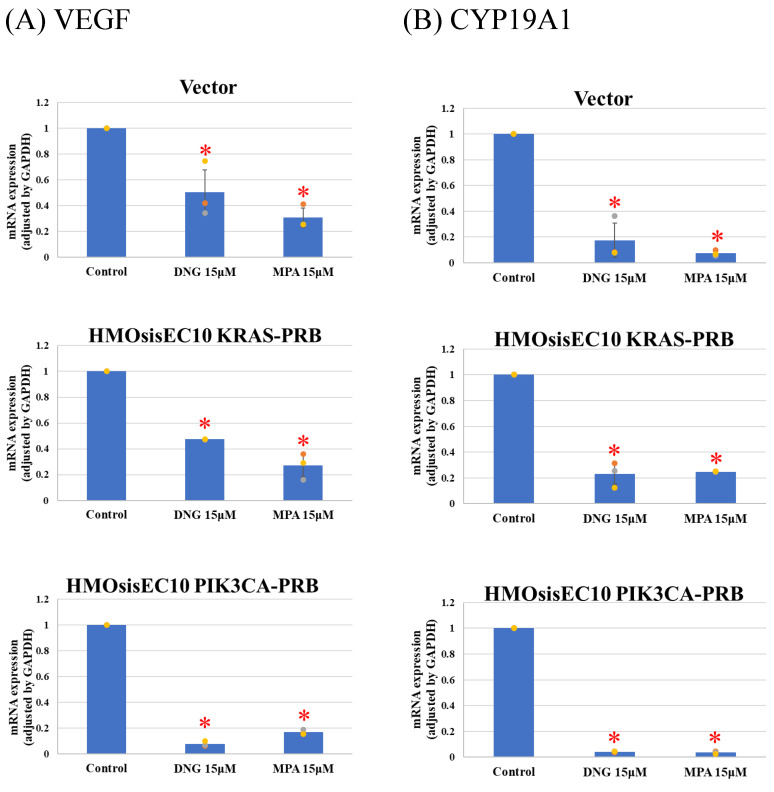
Real-Time quantitative PCR of (**A**) vascular endothelial growth factor (VEGF) and (**B**) cytochrome P 19A1 (CYP19A1) expression. Dienogest (DNG) and medroxyprogesterone (MPA) significantly suppressed VEGF and CYP19A1 expression in mutant cell lines. * *p* < 0.05 examined using Dunnett’s test. n = 3. The error bars indicate standard deviation.

Real-time qPCR of prostaglandin synthesis enzymes revealed significant suppression of Cox-2 and microsomal prostaglandin E2 (PGE2) synthases-1 (mPGES-1) in Vector and HMOsisEC10PIK3CA-PRB cells, whereas the *KRAS* mutated cell line was resistant to progesterone (Figure 5). All cell lines expressed IL-6 and MCP-1 after DNG or MPA treatment (Figure 6). However, expression of VEGF and CYP19A1 was suppressed in all cell lines in the presence of DNG or MPA (Figure 7).

## 4. Discussion

Endometriosis consists of endometrium-like glands and stroma outside the uterus [28], and usually occurs in the ovary, peritoneum of the Douglas area, the sacrouterine ligaments, and the gastrointestinal tract [9,28,29]. Endometriosis is classified by the revised American Society for Reproductive Medicine (rASRM), the Enzian classification, or the endometriosis fertility index (EFI). Concisely, it can be classified in superficial peritoneal endometriosis, ovarian endometriomas, and deep-infiltrating endometriosis (DIE) [30]. The major theory of pathogenesis of endometriosis is retrograde menstruation [31]; endometrial epithelial cells and stromal cells retrograde into the pelvic cavity through fallopian tubes and proliferate, adhere, and cause pain or infertility. It is also suggested that genetic factors and epigenetic factors are associated with endometriosis [28,32,33].

The major symptom of endometriosis is pain, such as dysmenorrhea, cyclic lower abdominal pain, chronic pelvic pain, dyspareunia, and painful defecation [1,9,12]. Physiologically, biochemicals such as PGE2 activate nociceptors and then the sensory nerve, and those signals are modulated at the spine and referred to the brain and the signal is recognized as “pain” [9,34,35]. Focusing on endometriosis-related pain, it is suggested that: (1) cyclic release of pain mediators and inflammatory mediators activate nociceptors resulting in dysmenorrhea and cyclic lower abdominal pain; (2) increased sensory nerve fibers and decreased sympathetic nerve fibers, and an imbalance of proinflammatory and anti-inflammatory sympathetic neurotransmitters, cause acyclic chronic pelvic pain (neurogenic inflammation); and (3) cyclic and repeated pain causes increase the nociceptive fields and lead to spinal hyperalgesia resulting in painful defecation [9,36,37]. The major therapies for endometriosis are low-dose estrogen progestin (LEP), progestins (DNG and MPA), gonadotropin-releasing hormone agonists, NSAIDs, and surgery [1,38]. Those differences of mechanisms of pain may result in NSAIDs and progestin therapy can ease dysmenorrhea and cyclic lower abdominal pain, while acyclic chronic pelvic pain or painful defecation cannot be removed enough by those therapy [9]. Unfortunately, endometriosis usually recurs even after surgery. The recurrence rates of endometriosis after two and five years are 19.1% and 20.5–43.5%, respectively [39], and postoperative progestin is used to prevent this recurrence [39,40]. Although DNG may reduce postoperative recurrence [41], 9% of patients with endometriosis do not respond to progestin therapy [42]. Progesterone resistance is reportedly due to the suppression of PR expression [14,43,44], PR signaling dysfunction [45], mesenchymal stem cells [46], and *KRAS* activation [47]. Suppression of PR expression is caused by suppression of estrogen receptor α (ERα) which increases PR expression [13,14,48], polymorphism [49], promoter hypermethylation [50] and microRNA dysregulation [13]. Increased *NOTCH1* [51,52] activity is associated with alterations in PR signaling, which suppresses PR activity. It is reported that *KRAS* mutation is related to hypermethylation on CpG islands in PR promoters, and therefore suppresses PR expression [53]. However, this suppression was observed in adenomyosis, not in endometriosis. Furthermore, AKT activity or increased MEK1/2 activity [45] is associated with altered PR signaling, which suppresses PR activity, and *KRAS* activation suppresses progesterone target genes such as Indian hedgehog (*IHH*) via sirtuin 1 (*SIRT1*) activation [47]. As described above, *KRAS* mutation and *PIK3CA* mutation may suppress PR activity by suppressing PR signaling and PR expression. Therefore, we hypothesized that endometrial cell lines harboring these cancer-related mutations are resistant to DNG and MPA. Hence, because our original HMOsisEC10 cell lines demonstrated inhibited expression of PR, we transduced PR to assess progesterone resistance. PR has two isoforms: PR-A, and PR-B [54]. PR-B has stronger transcriptional activation of progesterone target genes, whereas PR-A is a repressor of PR-B and other receptors [55,56,57]. Thus, since PR-B is the main progesterone receptor that suppresses endometriotic activity, we transduced PR-B into our cell lines. Our results showed significant suppression of cell migration, invasion, and proliferation. Bono et al. reported that even weak PR-B was responsive to progestin in an endometriotic cell line [25]. Therefore, even if the downstream PR is downregulated by *KRAS* or *PIK3CA* mutations, DNG and MPA may be sufficient to suppress endometriotic cell activity.

Since DNG suppresses pain- and inflammation-related factors, such as PGE2 [58,59], IL-6 [60], MCP-1 [60], VEGF [61], and CYP19A1 [58], to assess the impact of mutations on pain-related factors, we analyzed Cox-2 and mPGES-1 expression, as we could not obtain PEG2 primers. It is reported that COX-2 and mPGES-1 [62,63], which is involved in arachidonic acid cascade and synthesize PGE2, is elevated in endometriotic tissue [10]. PGE2 activates nociceptor sensory nerve endings, causing pain [64], and mediates inflammation [65]. Additionally, PGE2 is associated with direct angiogenesis [66], proliferation [67], adhesion [68], and invasion [69]. Vector and HMOsisEC10PIK3CA-PRB cells demonstrated inhibited expression of Cox-2 and mPGES-1 in the presence of progestins, whereas HMOsisEC10KRAS-PRB cells demonstrated resistance. Other investigated factors were progesterone sensitivity. Despite biophysiological effects of *KRAS* mutation on endometriotic cell are reported, little is known about clinical features and clinical progesterone response of *KRAS*-mutated endometriosis. It is reported that *KRAS* mutation is associated with greater anatomic disease burden and surgical complexity. Their results showed KRAS mutation is not associated with pain, but efficacy of progestin on pain is not assessed [70]. To the best of our knowledge, the mechanisms underlying *KRAS* mutation-associated progesterone resistance in PGE2 synthesis or pain in endometriotic epithelial cells are unknown. Our results suggest that even if *KRAS* mutations are not associated with progesterone resistance in endometriotic progression, they may be associated with progesterone resistance in endometriosis-induced pain. In previous research, we demonstrated that lysyl oxidase (LOX) and pentraxin 3 (PTX3) are upregulated in *KRAS* and *PIK3CA* mutated HMOsisEC10 cell lines as downstream targets. Interestingly, these inhibitions were experimentally proved to reduce cellular proliferative and invasive activity [24]. Thus, inhibition of *KRAS/PIK3CA*, or their downstream targets such as LOX or PTX3 inhibitors, might be clinically effective in progestin-resistant endometriosis-related pain.

This study has several limitations. First, our cell lines did not reflect mesenchymal function. Fibroblasts and mesenchymal stem cells are associated with progesterone resistance [46], and DNG inhibits endometriotic and endometrial stromal cell proliferation [71,72]. *NOTCH1* activation, which is associated with progesterone resistance, has been reported in endometriotic stromal cells, and is associated with decreased PR expression [51]. If we could have assessed not only endometriotic epithelial cells but also stromal cell functions, other findings may have been revealed. The use of xenograft models in future studies may solve this problem. Secondly, we transduced PR because PR expression was decreased in our cell line. Although PR expression in endometriotic epithelial cells is still controversial [15,73], there are two possible reasons for the decreased PR expression in our cell lines: PR expression was suppressed even in the original tumor, or suppressed during in vitro culture step or immortalization step [25]. If PR expression is suppressed during in vitro culture, an organoid culture system may solve this problem. Organoids are 3D culture systems that retain the biological and pathological features of the original tissue [74]. Organoid culture does not require immortalization, and it is expected that organoids can keep PR expression if original tumor expresses PR. In addition to solving immortalized process, organoid culture may solve the problem to assess epithelial-stromal crosstalk [75]. Thirdly, we used one cell line. In previous research, we established endometriotic epithelial cell lines with and without *KRAS* mutation or *PIK3CA* mutation. Our present research is based on those cell lines. Even the control is the same kind of cell lines. Using commercialized endometriotic epithelial cell line at least as a control could have made our data more reliable. We have ongoing project to establish further patient-derived endometriotic cell lines. Finally, DNG and MPA concentrations were significantly higher than their plasma concentrations. It has been reported that the mean maximum serum concentration of DNG is 6.8 × 10^−7^ M [76]. Previous studies have conducted experiments at concentration of 10^−7^ M [55,71]. This excessive concentration may conceal progesterone resistance, but it is notable that even under this concentration, *KRAS* mutation cell line showed progesterone resistant in terms of expression of Cox2 and mPGES-1.

## 5. Conclusions

In summary, we demonstrated that the immortalized human ovarian endometriotic cell line with *KRAS* or *PIK3CA* mutations are progesterone sensitive in migration, invasion, and proliferation. Our real-time quantitative PCR showed progesterone sensitive in IL-6, MCP-1, VEGF, and CYP19A1 expression, but it showed progesterone resistant in Cox-2 and mPGES-1 expression. They suggest that *KRAS* mutation and *PIK3CA* mutation may not be associated with progesterone resistance in terms of aggressiveness of endometriosis; however, progesterone resistance caused by *KRAS* mutations may affect pain.

## Data Availability

The data presented in this study are available on request from the corresponding author (K.N.).

## Supplementary Materials

The following supporting information can be downloaded at: https://www.mdpi.com/article/10.3390/cimb46040224/s1, Figure S1: Presentative fields of migration assay at 200× magnification; Figure S2: Presentative fields of invasion assay at 200× magnification; Table S1: Description of primary antibodies; Table S2: The primers used for real-time qPCR.
