# Supplementary material for: Association between KRAS and PIK3CA Mutations and Progesterone Resistance in Endometriotic Epithelial Cell Line
Source: Curr Issues Mol Biol. 2024 Apr 19;46(4):3579–94. doi: 10.3390/cimb46040224 (PMC11049223; doi:10.3390/cimb46040224)
Supplement: Supplementary file 1 [file cimb-46-00224-s001.zip › cimb-2949032-supplementary.pdf]

Association between *KRAS* or *PIK3CA* mutations and progesterone resistance on endometriotic epithelial cell line.

Supplementary materials

Table S1. Description of primary antibodies

Antibody	Manufacturer	Clone No.	Dilution	MW (kDa)
Progesterone Receptor A/B Antibody	Cell signaling	3176 (product No.)	1:1000	90 (PR-A)<br>118 (PR-B)
Progesterone Receptor B	Cell signaling	C1A2	1:1000	118
GAPDH	Wako	5A12	1:1000	37

Table S2. The primers used for real-time qPCR

Gene	Forward primer (5’ to 3’)	Reverse primer (3’ to 5’)
COX-2	CGGTGAAACTCTGGCTAGACAG	GCAAACCGTAGATGCTCAGGGA
mPGES-1	GGAACGACATGGAGACCATCTAC	TCCAG GCGACAAAAGGGTTA
IL-6	AGACAGCCACTCACCTCTTCAG	TTCTGCCAGTGCCTCTTTGCTG
MCP-1	AGAATCACCAGCAGCAAGTGTCC	TCCTGAACCCACTTCTGCTTGG
VEGF	TGCAGATTATGCGGATCAAACC	TGCATTCACATTTGTTGTGCTGTAG
CYP19A1	GACGCAGGATTTCCACAGAAGAG	ATGGTGTCAGGAGCTGCGATCA
GAPDH	TGTTGCCATCAATGACCCCTT	CTCCACGACGTACTCAGCG

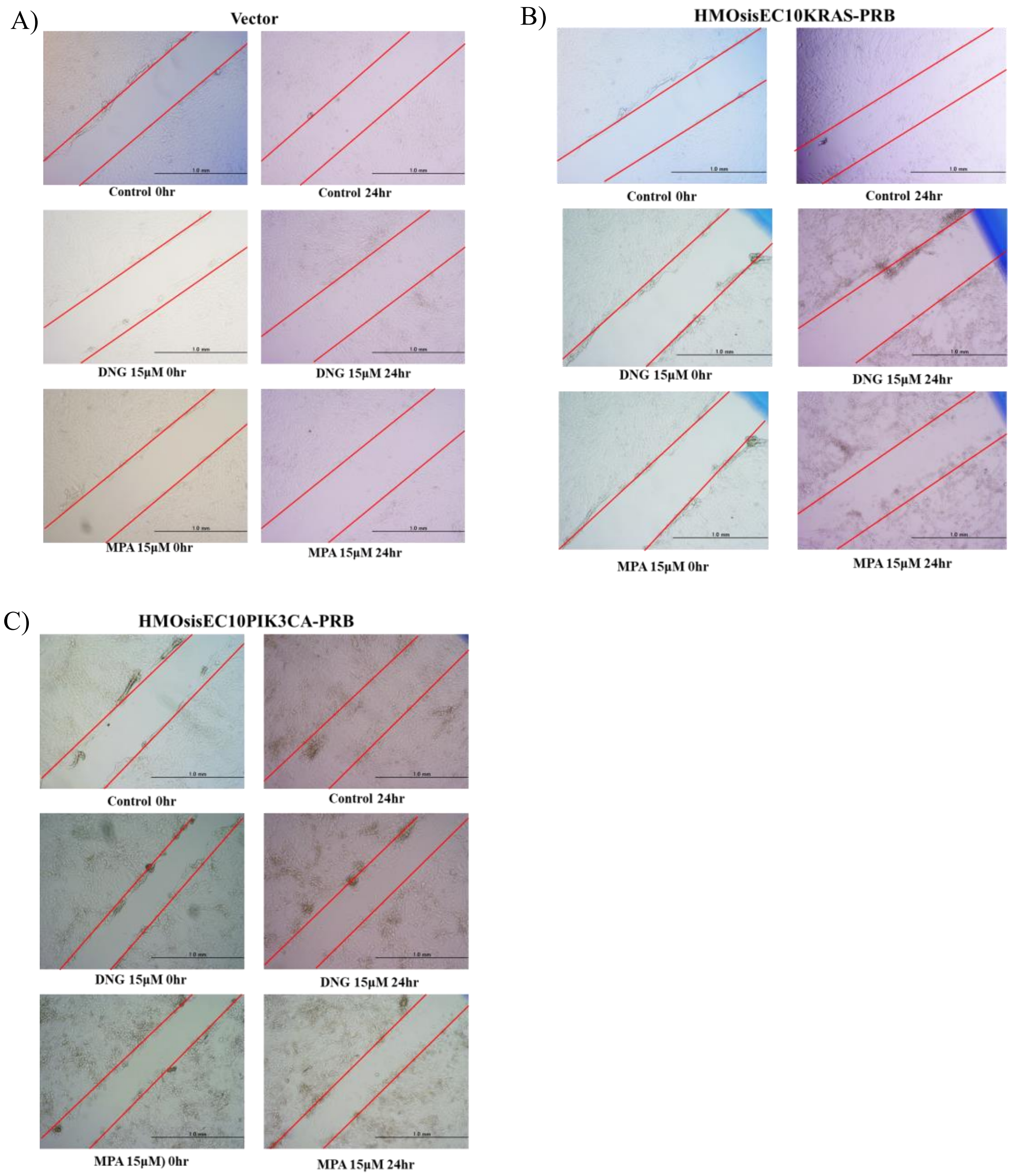

Figure S1. Presentative fields of migration assay at 200× magnification.

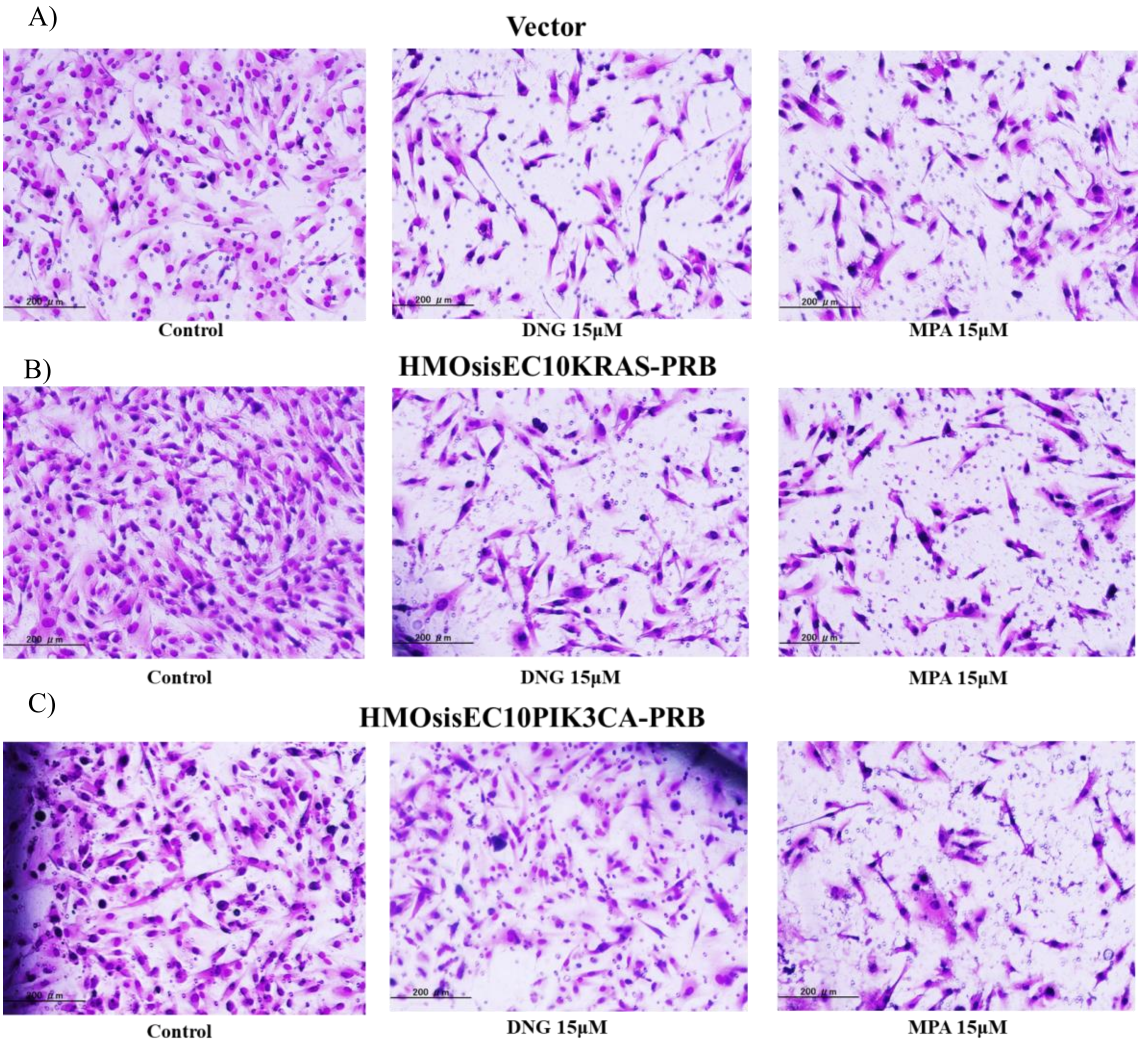

Figure S2. Presentative fields of invasion assay at 200× magnification.
